# Soil Depth Influences Fungal Community Structure and Ecological Processes in a Degraded Soda Saline–Alkali Wetland

**DOI:** 10.3390/biology15120911

**Published:** 2026-06-10

**Authors:** Junnan Ding, Xin Li

**Affiliations:** 1 Heilongjiang Province Key Laboratory of Cold Region Wetland Ecology and Environment Research, Harbin University, Harbin 150086, China; 2 College of Resources and Environment, Northeast Agricultural University, Harbin 150030, China; swx05256lx@126.com

**Keywords:** subsurface soil, salt-affected soils, edaphic heterogeneity, stochastic processes, trophic modes

## Abstract

Soil depth and habitat degradation can reshape fungal communities in salt-affected wetlands, but their effects on fungal ecological processes remain insufficiently understood. This study examined soil fungi in the Halahai Provincial Nature Reserve and adjacent converted farmland in the western Songnen Plain, Northeast China, where salt-affected meadow soils correspond mainly to Solonetz. Four habitat types—reed wetland, meadow steppe, degraded *Suaeda* saline patch, and converted farmland—were sampled at 0–20 cm and 20–40 cm soil depths. Soil properties, fungal diversity, taxonomic composition, environmental associations, niche breadth, assembly processes, and FUNGuild-based trophic modes were analyzed using ITS sequencing. Degraded Suaeda soils showed the strongest salinity–alkalinity stress, with pH values of 10.34–10.30 and electrical conductivity of 1.70–1.75 dS·m^−1^. Fungal richness was highest in surface-converted farmland, with a Sobs value of 423.33, and lowest in deeper degraded Suaeda soil, with a Sobs value of 86.00. Ascomycota dominated most groups, especially degraded Suaeda soils, where its relative abundance reached 75.29–76.80%. ANOSIM confirmed significant community dissimilarity among habitat-depth groups (R = 0.56878, *p* = 0.001). Specialists accounted for 68.07% of fungal taxa, and stochastic processes, especially drift and dispersal limitation, contributed substantially to assembly. These results indicate that soil depth, salinity–alkalinity, and habitat conversion jointly regulate fungal community structure and ecological processes in degraded soda saline–alkali wetlands.

## Simple Summary

Salt-affected wetlands are fragile ecosystems, and their degradation can strongly change soil conditions and belowground organisms. This study examined soil fungi in the Halahai Provincial Nature Reserve and adjacent converted farmland in Qiqihar, Northeast China, where the soils are salt-affected meadow soils corresponding mainly to Solonetz under international soil classification standards. We compared four habitat types and two soil layers, 0–20 cm and 20–40 cm, to determine how wetland degradation, farmland conversion, and soil depth influence fungal communities. Degraded saline patches had the strongest soil stress, with higher alkalinity and salinity but lower water content, carbon, nutrients, and microbial biomass. Fungal richness was highest in surface-converted farmland soil, with an observed richness of 423.33, and lowest in deeper degraded saline soil, with an observed richness of 86.00. Ascomycota dominated most treatments, especially degraded saline soils, where its relative abundance reached 75.29–76.80%. Specialist fungal taxa accounted for 68.07%, while generalists accounted for 18.48%. These results show that soil depth and salinity–alkalinity jointly influence fungal community structure and potential ecological roles, providing useful information for restoring and managing degraded salt-affected wetlands.

## 1. Introduction

Wetland soils often exhibit strong vertical heterogeneity in physicochemical conditions and microbial habitats. Even within a relatively shallow soil profile, water availability, aeration, salt accumulation, organic matter input, root distribution, and nutrient turnover can change markedly with depth [1,2]. These vertical changes are particularly important in saline–alkali wetlands, where hydrological fluctuation and strong evaporation can redistribute salts and nutrients between surface and deeper soil layers [3]. Consequently, microorganisms inhabiting different soil depths may experience contrasting environmental constraints, even within the same wetland landscape [4]. Understanding this vertical structure is essential for evaluating belowground biological responses to wetland degradation, because surface soil alone may not fully represent microbial diversity, community composition, ecological strategies, or assembly processes.

Soda saline–alkali wetlands in Northeast China provide a representative system for examining soil-depth-dependent microbial responses. In these wetlands, degradation is often accompanied by reduced water connectivity, intensified salinity–alkalinity, vegetation replacement, soil compaction, and declining soil fertility [5,6]. Previous studies in the Songnen Plain and other saline wetlands have shown that soil pH, salinity, moisture, organic carbon, nutrient availability, and enzyme activities can strongly shape microbial diversity and community composition [7,8,9]. However, many studies have focused mainly on horizontal habitat gradients, degradation stages, or land-use transitions, while the vertical distribution of microbial communities within the soil profile remains less well understood [10]. This limitation is important because soil depth may alter not only fungal diversity and taxonomic composition, but also the relative importance of ecological assembly processes and potential functional guilds.

Soil fungi are key regulators of organic matter decomposition, nutrient transformation, plant residue turnover, and plant–soil interactions [11,12]. Compared with bacteria, fungi often differ in dispersal strategies, hyphal growth, substrate use, stress tolerance, and dependence on plant-derived carbon inputs [13]. These traits may allow some fungal groups to persist under fluctuating moisture, salinity, or nutrient limitation, while also making fungal communities sensitive to changes in root distribution, organic matter quality, and soil physicochemical conditions along depth gradients [14]. Surface soils are usually more strongly influenced by plant litter, fine roots, and labile carbon inputs, whereas deeper soils generally contain fewer fresh substrates and may impose stronger constraints through lower nutrient availability, altered aeration, and salt accumulation [15,16]. Therefore, fungal communities in soda saline–alkali wetlands may exhibit clear habitat- and depth-dependent differentiation, but the ecological processes underlying this differentiation remain insufficiently resolved.

Two complementary perspectives can be considered. First, fungal communities may respond to soil depth and habitat gradients through taxonomic turnover, with dominant fungal groups and indicator taxa changing along salinity–alkalinity and nutrient gradients [17]. Second, such taxonomic shifts may be accompanied by changes in ecological strategies and community assembly processes. For example, specialist taxa with narrow niche breadths may increase under strong environmental heterogeneity, whereas generalist taxa may persist across broader habitat-depth gradients [18]. At the same time, fungal community assembly may be shaped by both deterministic processes, such as environmental selection, and stochastic processes, such as ecological drift and dispersal limitation. Distinguishing these processes is important for understanding whether fungal communities are primarily structured by edaphic filtering or by stochastic ecological processes in degraded saline–alkali wetlands.

Functional interpretation is also needed because taxonomic shifts alone do not indicate whether fungal community changes are linked to decomposition, symbiosis, pathogenicity, or mixed trophic strategies. Fungal functional guild analysis provides a useful, although predictive, approach for linking taxonomic composition with potential ecological roles, including saprotrophic, symbiotic, pathogenic, and mixed trophic guilds [19]. In saline–alkali wetlands, variation in saprotrophic fungi may reflect changes in organic matter decomposition potential, while shifts in plant-associated or pathogenic guilds may indicate changes in plant–soil interactions under degradation or agricultural conversion [20]. However, few studies have jointly considered soil depth, habitat degradation, agricultural conversion, fungal taxonomic turnover, ecological assembly processes, niche breadth, and predicted functional guilds within the same soda saline–alkali wetland system. This integrated perspective is important for clarifying whether fungal community changes are mainly associated with vertical soil heterogeneity, salinity–alkalinity stress, nutrient limitation, or their combined effects.

In this study, we investigated soil fungal communities at two depths, 0–20 cm and 20–40 cm, across four habitat types in the Halahai Provincial Nature Reserve and adjacent converted farmland in the western Songnen Plain, Northeast China. The selected habitats included reed wetland, meadow steppe, degraded Suaeda saline patch, and converted farmland, representing wetland degradation and agricultural conversion gradients. The originality of this study lies in its integrated evaluation of fungal diversity, taxonomic composition, differential indicator taxa, beta diversity, environmental associations, niche breadth, ecological assembly processes, and FUNGuild-based functional guilds along both habitat and soil-depth gradients. The specific aims were to: (1) determine how fungal diversity and community composition vary among habitat-depth groups; (2) identify fungal indicator taxa and soil environmental factors associated with community differentiation; (3) evaluate fungal ecological strategies and assembly processes across habitat-depth gradients; and (4) assess whether predicted fungal functional guilds differ among habitats and soil depths. We hypothesized that degraded saline patches and deeper soils would impose stronger edaphic constraints on fungal communities, leading to lower richness, distinct taxonomic composition, a higher contribution of specialist taxa, and altered assembly processes and functional guilds. By integrating community composition, ecological assembly, and predicted functional traits, this study aims to clarify how soil depth mediates fungal community responses to degradation and conversion in soda saline–alkali wetlands.

## 2. Materials and Methods

### 2.1. Study Area

**Figure 1:**
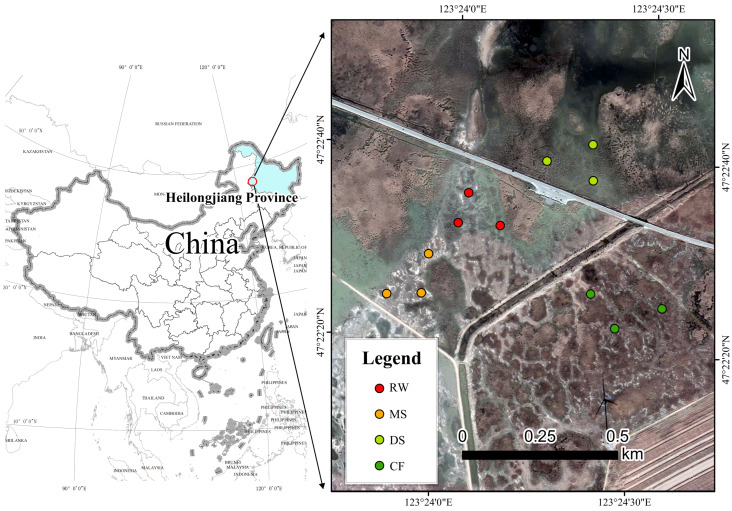
Study area and sampling sites. RW, reed wetland; MS, meadow steppe; DS, degraded *Suaeda* saline patch; CF, converted farmland. Numbers indicate three replicate sampling sites within each habitat type. At each site, soil samples were collected from two depths: 0–20 cm and 20–40 cm.

The study was conducted in the Halahai Provincial Nature Reserve and adjacent converted farmland in Qiqihar City, Heilongjiang Province, Northeast China (47°20′–47°34′ N, 124°10′–124°32′ E). This region, located in the western Songnen Plain, represents a typical soda saline–alkali wetland characterized by saline meadow soils and alkaline meadow soils. Salt-affected soils here are highly alkaline and soda-rich, and correspond mainly to Solonetz according to international soil classification standards [21]. The climate is temperate continental monsoon, with cold winters, warm summers, concentrated growing-season precipitation, and annual evaporation generally exceeding precipitation. Four habitat types were selected for sampling: reed wetland (RW), meadow steppe (MS), degraded *Suaeda* saline patch (DS), and converted farmland (CF). RW was dominated by *Phragmites australis* and other hygrophytic vegetation; MS by meadow grasses such as *Leymus chinensis* and *Calamagrostis epigejos*; DS by sparse halophytic plants, mainly *Suaeda glauca*; and CF by maize (*Zea mays*) farmland converted from former wetland. RW, MS, and DS represent a natural wetland degradation gradient, while CF represents agricultural conversion. The distribution of sampling sites is shown in Figure 1 [22]. Each habitat was sampled at two soil depths, 0–20 cm and 20–40 cm. The corresponding habitat-depth groups were coded as RW1, RW2, MS1, MS2, DS1, DS2, CF1, and CF2, where “1” and “2” indicate the 0–20 cm and 20–40 cm soil layers, respectively.

### 2.2. Sampling Sites and Experimental Design

Based on the four habitat types described above, three independent 10 m × 10 m plots were established in each habitat as biological replicates. The plots were located in relatively homogeneous areas and separated by sufficient distance to reduce spatial dependence. Within each plot, soil samples were collected from two depth intervals, 0–20 cm and 20–40 cm. For each depth, five soil cores were taken from the center and four corners of the plot using a sterile stainless-steel auger and then mixed to form one composite sample. In total, 24 composite soil samples were obtained, corresponding to four habitat types, two soil depths, and three biological replicates. Visible roots, litter, stones, and coarse debris were removed manually. The auger was cleaned with 75% ethanol between plots and depths to avoid cross-contamination. Each composite sample was divided into three subsamples: one stored at −80 °C for deoxyribonucleic acid (DNA) extraction, one stored at 4 °C for enzyme activity and available nutrient analyses, and one air-dried for soil physicochemical measurements.

### 2.3. Soil Physicochemical Properties and Enzyme Activity Assays

Soil water content (SWC) was measured gravimetrically by drying fresh soil samples at 105 °C to constant weight [23]. Bulk density (BD) was determined using the core method after oven-drying undisturbed soil samples [24]. Soil pH was measured in a soil–deionized water suspension at a soil-to-water ratio of 1:2.5 (*w*/*v*) using a pH meter (Bante Instruments Co., Ltd., Shanghai, China) and is reported as pH (H_2_O) [25], and electrical conductivity (EC) was determined in a 1:5 soil-to-water extract with a conductivity meter (INESA Scientific Instrument Co., Ltd., Shanghai, China) [26]. Soil organic carbon (SOC) was analyzed using the dichromate oxidation method [27]. Dissolved organic carbon (DOC) was extracted from fresh soil and quantified using a total organic carbon analyzer (Shimadzu Corporation, Kyoto, Japan) [28]. Microbial biomass carbon (MBC) and microbial biomass nitrogen (MBN) were measured using the chloroform fumigation–extraction method [29]. Available nitrogen (AN) was determined as alkali-hydrolyzable nitrogen using the alkaline hydrolysis diffusion method [30]. Available phosphorus (AP) was extracted with 0.5 mol L^−1^ NaHCO_3_ and quantified colorimetrically [31]. Urease activity (URE) was used as an indicator of soil nitrogen transformation potential and was determined by incubating soil with urea substrate and quantifying the released ammonium colorimetrically [32]. Alkaline phosphatase activity (ALP) was used as an indicator of soil phosphorus transformation potential and was assayed using p-nitrophenyl phosphate as the substrate under alkaline conditions; the released p-nitrophenol was measured colorimetrically [33]. Both enzyme activities were expressed on a dry soil mass basis.

### 2.4. DNA Extraction, PCR Amplification, and ITS Sequencing

Total genomic deoxyribonucleic acid (DNA) was extracted from 0.5 g of fresh soil using the E.Z.N.A.^®^ Soil DNA Kit (Omega Bio-tek, Norcross, GA, USA), following the manufacturer’s instructions. The concentration and purity of the extracted DNA were evaluated using a NanoDrop 2000 spectrophotometer (Thermo Fisher Scientific, Wilmington, DE, USA), and DNA integrity was checked by 1% agarose gel electrophoresis. The fungal internal transcribed spacer 2 (ITS2) region was amplified using the primers ITS3, 5′-GCATCGATGAAGAACGCAGC-3′, and ITS4, 5′-TCCTCCGCTTATTGATATGC-3′. Polymerase chain reaction (PCR) amplification was performed in triplicate for each sample to reduce amplification bias. The PCR program included an initial denaturation at 95 °C for 3 min, followed by 25 cycles of denaturation at 95 °C for 30 s, annealing at 55 °C for 30 s, and extension at 72 °C for 45 s, with a final extension at 72 °C for 10 min. The PCR products were examined by agarose gel electrophoresis, purified using a commercial purification kit (Omega Bio-Tek, Norcross, GA, USA), and quantified using a fluorometric method. Purified amplicons were pooled in equimolar concentrations and subjected to paired-end sequencing on an Illumina platform at Majorbio Bio-Pharm Technology Co., Ltd. (Shanghai, China). The project accession number PRJNA1472827 has been assigned to the sequences stored in NCBI’s SRA database.

### 2.5. Sequence Processing and Taxonomic Annotation

Raw paired-end reads were processed on the Majorbio Cloud Platform. Low-quality reads, reads containing ambiguous bases, and sequences shorter than the required length were removed before downstream analysis. Paired-end reads were merged according to their overlapping regions using Fast Length Adjustment of Short reads (FLASH) [34]. Chimeric sequences were identified and discarded to obtain high-quality fungal internal transcribed spacer (ITS) sequences. The high-quality sequences were clustered into operational taxonomic units (OTUs) at 97% sequence similarity using UPARSE software (version 7.1) [35]. Representative sequences from each OTU were selected for taxonomic annotation against the UNITE fungal database [36]. Sequences assigned to non-fungal taxa, chloroplasts, mitochondria, or other non-target groups were removed before subsequent analyses. To reduce the influence of unequal sequencing depth among samples, all samples were rarefied to the same sequencing depth before diversity analysis. The final OTU abundance table was used for fungal alpha diversity, beta diversity, taxonomic composition, LEfSe analysis, environmental correlation analysis, ecological assembly analysis, niche breadth analysis, and FUNGuild-based functional guild prediction.

### 2.6. Fungal Diversity, Taxonomic Composition, and Differential Taxa Analyses

Fungal alpha diversity was evaluated using the Sobs, ACE, Shannon, and Simpson indices. Sobs was used to represent observed richness, ACE was used to estimate fungal richness, and Shannon and Simpson indices were used to evaluate fungal community diversity and dominance [37]. Fungal taxonomic composition was summarized at the phylum and class levels based on the final OTU abundance table. Taxa with low relative abundance or unresolved taxonomic assignment were grouped as “others” for visualization. Shared and unique fungal taxa among habitat types and habitat-depth groups were displayed using Venn diagrams. Beta diversity was calculated based on Bray–Curtis dissimilarity [38]. Principal coordinate analysis (PCoA) and non-metric multidimensional scaling (NMDS) were used to visualize differences in fungal community structure among habitat-depth groups. Analysis of similarities (ANOSIM) was used to test whether fungal community composition differed significantly among groups [39]. Differential fungal taxa among habitat-depth groups were identified using linear discriminant analysis effect size (LEfSe) [40]. Linear discriminant analysis (LDA) scores were used to identify the taxa that contributed most strongly to differences among habitat-depth groups.

### 2.7. Environmental Correlation and Functional Guild Analyses

Relationships between fungal communities and soil environmental variables were evaluated using constrained ordination and correlation analyses. Detrended correspondence analysis (DCA) was first conducted to determine the appropriate constrained ordination method, after which canonical correspondence analysis (CCA) was used to assess the associations between fungal community composition and soil variables [41]. Soil variables included pH, EC, SWC, SOC, BD, AN, AP, DOC, MBC, MBN, URE, and ALP. Spearman correlation analysis was used to examine relationships between dominant fungal phyla and soil environmental variables [42]. Predicted fungal functional guilds were assigned using FUNGuild based on fungal taxonomic information [43]. OTUs were classified into functional guilds and trophic categories, including saprotrophic, pathogenic, symbiotic, and mixed trophic groups. Taxa without a reliable functional assignment were retained as “unknown”. Because FUNGuild predictions are based on taxonomic annotation rather than direct functional measurements, the results were interpreted as predicted functional tendencies [44].

### 2.8. Ecological Assembly and Statistical Analysis

Fungal ecological strategies were evaluated using niche breadth analysis based on OTU distribution patterns across samples. Fungal taxa were classified as generalists, neutralists, or specialists according to their niche breadth values. Community assembly processes were assessed using the beta nearest taxon index (βNTI) and iCAMP analysis. βNTI was used to estimate the relative importance of deterministic and stochastic processes, while iCAMP was applied to further partition community assembly into homogeneous selection, heterogeneous selection, dispersal limitation, homogeneous dispersal, and drift or other undominated processes [45]. A neutral community model (NCM) was used to evaluate the relationship between OTU occurrence frequency and mean relative abundance and to estimate the potential contribution of neutral processes to fungal community distribution [46]. All statistical analyses were performed in R and on the Majorbio Cloud Platform. Each habitat-depth group contained three biological replicates. Differences in soil properties, enzyme activities, fungal alpha diversity indices, and predicted functional guilds among habitat-depth groups were tested using one-way analysis of variance (ANOVA), followed by Tukey’s post hoc test when appropriate [47]. When multiple comparisons were performed, *p*-values were adjusted using the false discovery rate (FDR) method [48]. Statistical significance was defined at *p* < 0.05.

## 3. Results

### 3.1. Soil Physicochemical Properties Across Soil Depths

As shown in Table 1, soil physicochemical properties, microbial biomass, and enzyme activities varied markedly among habitat-depth groups. Soil pH was alkaline across all treatments, with the highest values observed in DS1 and DS2, at 10.34 and 10.30, respectively, which were significantly higher than those in the other groups; the remaining treatments ranged from 8.47 to 8.81. EC reached its highest values in DS1 and DS2, at 1.70 and 1.75 dS·m^−1^, respectively, and was significantly higher than that in the RW, MS, and CF groups, with CF2 showing a higher EC than CF1. SWC was higher in RW1 and RW2, at 46.50% and 40.50%, respectively, followed by CF2 at 32.17%, whereas DS1 and DS2 had lower values of 12.83% and 14.83%, respectively. SOC was highest in RW1, reaching 26.17 g·kg^−1^, while RW2, MS1, and CF1 showed intermediate values; DS1 and DS2 had lower SOC contents, with the minimum value recorded in DS2 at 4.27 g·kg^−1^. BD was highest in CF2, at 1.57 g·cm^−3^, and remained relatively high in CF1, DS1, and DS2, whereas RW2 had the lowest value of 0.85 g·cm^−3^. AN was higher in CF1 and RW1, at 225.50 and 192.73 mg·kg^−1^, respectively, and was markedly lower in DS1 and DS2. AP reached its maximum in CF1, at 28.40 mg kg^−1^, and its minimum in DS2, at 2.60 mg·kg^−1^. DOC was higher in RW1 and MS1, with values of 388.68 and 370.31 mg·kg^−1^, respectively, and lower in DS1 and DS2. MBC was highest in RW1, at 1865.06 mg·kg^−1^, followed by RW2 and MS1, and lowest in DS2. MBN was highest in both RW1 and RW2, at 256.11 mg·kg^−1^, significantly exceeding the other treatments, whereas DS2 had the lowest value of 46.13 mg·kg^−1^. URE showed the highest value in RW1, at 102.08 mg·g^−1^, and the lowest value in DS2, at 22.51 mg·g^−1^. ALP was highest in DS2, reaching 217.90 mg·g^−1^, with relatively high values also observed in CF1, RW1, and DS1, while RW2, MS1, and CF2 showed comparatively lower activities.

### 3.2. Alpha Diversity of Fungal Communities Across Soil Depths

As shown in Table 2, fungal alpha diversity indices varied among the habitat-depth groups. The Sobs index was highest in CF1, reaching 423.33, and was significantly higher than that in the other treatments; CF2 showed the second-highest value, at 267.00. Lower Sobs values were observed in DS1 and DS2, at 110.33 and 86.00, respectively, with DS2 showing the lowest value. The Sobs values in RW1, RW2, MS1, and MS2 were intermediate, ranging from 155.00 to 208.67. The Shannon and Simpson indices showed numerical variation among habitat-depth groups, but no significant differences were detected among treatments according to the significance letters. The highest Shannon value was observed in CF1, whereas DS1 and DS2 showed relatively lower values. The Ace index showed a pattern similar to that of Sobs, with the highest value recorded in CF1, at 622.76, followed by CF2 at 274.57. The RW and MS groups ranged from 156.96 to 212.37, whereas DS1 and DS2 showed lower values of 115.35 and 87.24, respectively. The Simpson index was numerically higher in CF1, DS1, and CF2 and lowest in MS1. In contrast, Sobs and Ace differed markedly among habitat-depth groups, indicating that fungal richness was more sensitive than community diversity or dominance to habitat-depth variation.

### 3.3. Soil Depth-Dependent Fungal Taxonomic Composition

**Figure 2:**
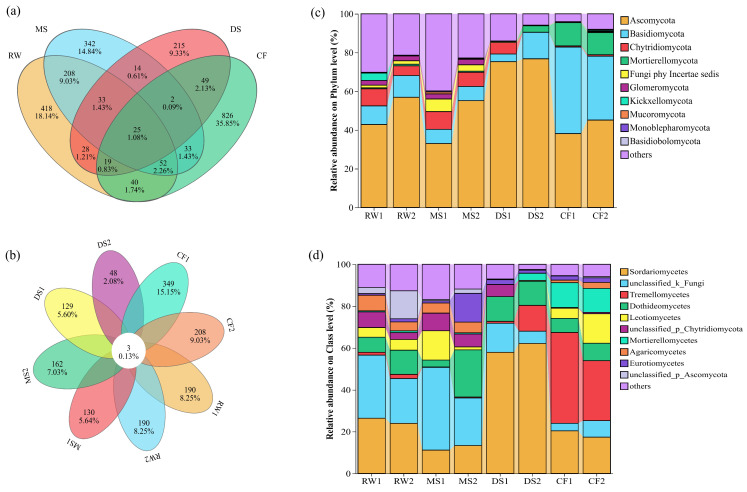
Soil depth-dependent fungal taxonomic composition across different habitat types in a degraded soda saline–alkali wetland. (**a**) Venn diagram showing the shared and unique fungal taxa among the four habitat types. (**b**) Venn diagram showing the shared and unique fungal taxa among the eight habitat-depth groups. (**c**) Relative abundance of dominant fungal phyla across the eight habitat-depth groups. (**d**) Relative abundance of dominant fungal classes across the eight habitat-depth groups. RW, reed wetland; MS, meadow steppe; DS, degraded *Suaeda* saline patch; CF, converted farmland; 1, 0–20 cm soil layer; 2, 20–40 cm soil layer.

As shown in Figure 2, fungal taxonomic composition varied across habitat types and soil depths. The Venn analysis showed that 25 fungal taxa were shared among RW, MS, DS, and CF, accounting for 1.08% of the total taxa, whereas CF had the highest number of unique taxa, with 826 taxa accounting for 35.85% (Figure 2a). When separated by habitat-depth groups, only 3 taxa were shared by all eight groups, accounting for 0.13% (Figure 2b). CF1 contained the largest number of unique taxa, followed by CF2, whereas DS2 had the fewest unique taxa. At the phylum level, Ascomycota was dominant in most groups, particularly in DS1 and DS2, where its relative abundance reached 75.29% and 76.80%, respectively (Figure 2c). The relative abundance of Ascomycota increased with soil depth in RW and MS, while Basidiomycota was highly abundant in CF, especially in CF1. Mortierellomycota was mainly enriched in CF1 and CF2, whereas Chytridiomycota showed relatively higher proportions in RW1, MS1, MS2, and DS1 (Figure 2c). At the class level, Sordariomycetes was dominant in DS1 and DS2 and also occurred widely in the other groups. Tremellomycetes were mainly enriched in CF1 and CF2, while unclassified fungi at the kingdom level were more abundant in RW1 and MS1. Dothideomycetes, Leotiomycetes, unclassified Chytridiomycota, Mortierellomycetes, Agaricomycetes, and Eurotiomycetes also showed habitat- and depth-dependent variation (Figure 2d).

### 3.4. Differential Fungal Taxa Across Habitat-Depth Groups Identified by LEfSe

**Figure 3:**
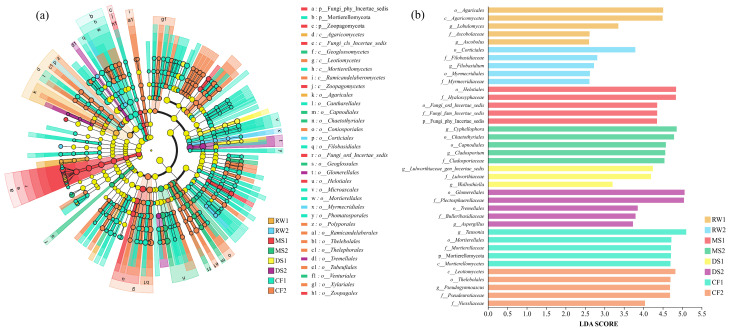
LEfSe analysis of differential fungal taxa across habitat-depth groups. (**a**) LEfSe cladogram showing differential fungal taxa among the eight habitat-depth groups. (**b**) LDA score plot showing the discriminant fungal taxa with significant differences among groups. Different colors indicate taxa enriched in different habitat-depth groups. LEfSe, linear discriminant analysis effect size; LDA, linear discriminant analysis; RW, reed wetland; MS, meadow steppe; DS, degraded *Suaeda* saline patch; CF, converted farmland; 1, 0–20 cm soil layer; 2, 20–40 cm soil layer.

LEfSe analysis identified distinct fungal biomarkers among the eight habitat-depth groups (Figure 3). The cladogram showed that the differential taxa were mainly distributed from the phylum to order levels, indicating clear taxonomic differentiation among habitat-depth groups (Figure 3a). At the phylum level, Fungi_phy_Incertae_sedis and Zoopagomycota were mainly associated with MS1, whereas Mortierellomycota was enriched in CF1. At the class and order levels, RW1 was characterized by *Agaricomycetes* and *Agaricales*, while RW2 was associated with *Corticiales* and *Myrmecridiales*. MS1 was mainly represented by *Helotiales*, *Fungi_ord_Incertae_sedis*, and *Zoopagales*, whereas MS2 was associated with *Chaetothyriales*, *Capnodiales*, *Geoglossales*, and *Venturiales*. DS2 showed enrichment of *Glomerellales* and *Tremellales*. In the converted farmland groups, CF1 was mainly characterized by *Mortierellales*, *Filobasidiales*, *Microascales*, Cantharellales, and *Phomatosporales*, whereas CF2 was associated with *Thelebolales*, *Tubeufiales*, *Thelephorales*, *Xylariales*, *Ramicandelaberales*, *Coniosporiales*, and *Polyporales*. The LDA score plot further showed that the major discriminant taxa differed among groups (Figure 3b). MS1 was mainly distinguished by unclassified fungal lineages, while CF1 showed high LDA scores for *Tausonia*, *Mortierellomycota*, *Mortierellomycetes*, *Mortierellales*, and *Mortierellaceae*. DS2 was mainly characterized by *Glomerellales*, *Plectosphaerellaceae*, *Tremellales*, *Bulleribasidiaceae*, and *Aspergillus*. MS2 was distinguished by *Cyphellophora*, *Chaetothyriales*, *Capnodiales*, *Cladosporium*, and *Cladosporiaceae*, whereas CF2 was mainly associated with *Leotiomycetes*, *Thelebolales*, *Pseudogymnoascus*, *Pseudeurotiaceae*, *Niessliaceae*, and *Tubeufiales*. RW1 and RW2 were mainly differentiated by *Agaricomycetes*/*Agaricales* and *Corticiales-related* taxa, respectively.

### 3.5. Beta Diversity and Community Dissimilarity of Fungal Communities

**Figure 4:**
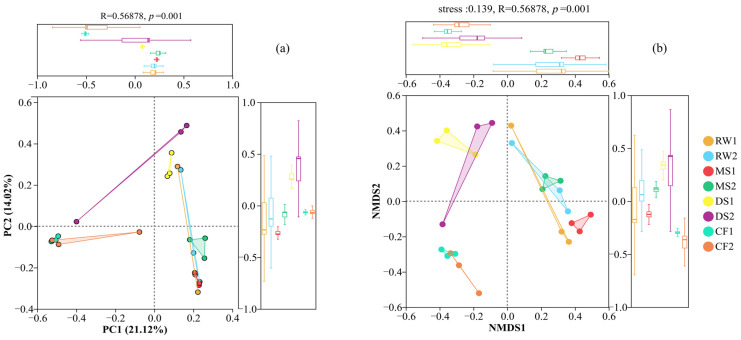
Beta diversity and community dissimilarity of fungal communities across habitat-depth groups. (**a**) PCoA and (**b**) NMDS ordinations showing differences in fungal community structure among the eight habitat-depth groups. ANOSIM indicated significant group differences. RW, reed wetland; MS, meadow steppe; DS, degraded *Suaeda* saline patch; CF, converted farmland; 1, 0–20 cm; 2, 20–40 cm.

As shown in Figure 4, both PCoA and NMDS analyses revealed clear differences in fungal community structure among the habitat-depth groups. In the PCoA ordination, PC1 and PC2 explained 21.12% and 14.02% of the total community variation, respectively, with a cumulative contribution of 35.14%. The NMDS stress value was 0.139, indicating that the ordination reliably represented the dissimilarities among samples. ANOSIM further confirmed significant differences in fungal community composition among treatments (R = 0.56878, *p* = 0.001). In the ordination space, CF1 and CF2 were clearly separated from the RW, MS, and DS groups, while DS1 and DS2 were mainly distributed in the upper region of the ordination plots, with DS2 showing relatively greater within-group dispersion. Samples from RW and MS were positioned relatively close to each other, although separation between soil depths was still observed.

### 3.6. Associations Between Fungal Community Structure and Soil Environmental Factors

**Figure 5:**
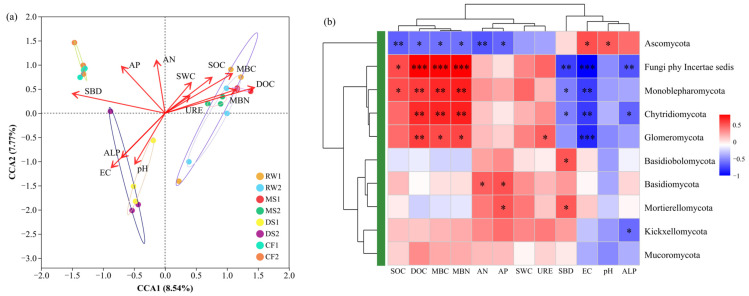
Associations between fungal community composition and soil environmental factors. (**a**) Canonical correspondence analysis (CCA) showing the relationships between fungal community structure and soil environmental factors. (**b**) Spearman correlation heatmap between dominant fungal phyla and soil variables. Red and blue indicate positive and negative correlations, respectively. RW, reed wetland; MS, meadow steppe; DS, degraded *Suaeda* saline patch; CF, converted farmland; 1, 0–20 cm soil layer; 2, 20–40 cm soil layer; SOC, soil organic carbon; DOC, dissolved organic carbon; MBC, microbial biomass carbon; MBN, microbial biomass nitrogen; AN, available nitrogen; AP, available phosphorus; SWC, soil water content; URE, urease activity; SBD, soil bulk density; EC, electrical conductivity; ALP, alkaline phosphatase activity. *, *p* < 0.05; **, *p* < 0.01; ***, *p* < 0.001.

As shown in Figure 5, the CCA ordination and Spearman correlation analysis revealed clear associations between fungal community composition and soil environmental factors. CCA1 and CCA2 explained 8.54% and 7.77% of the community variation, respectively, with a cumulative contribution of 16.31%. In the ordination plot, the habitat-depth groups showed separation along environmental gradients, and pH, EC, BD, SWC, SOC, DOC, MBC, MBN, AN, AP, URE, and ALP were associated with sample distribution to varying degrees. The correlation heatmap further showed distinct relationships between fungal phyla and soil variables. Ascomycota was mostly negatively correlated with SOC, DOC, MBC, MBN, AN, and AP, but positively correlated with EC and pH. Fungi phy Incertae sedis, Monoblepharomycota, Chytridiomycota, and Glomeromycota were generally positively correlated with DOC, MBC, and MBN and negatively correlated with EC or BD. Basidiomycota was positively correlated with AN and AP, while Mortierellomycota showed positive correlations with AP and BD. Kickxellomycota was negatively correlated with ALP.

### 3.7. Fungal Ecological Strategies and Community Assembly Processes

**Figure 6:**
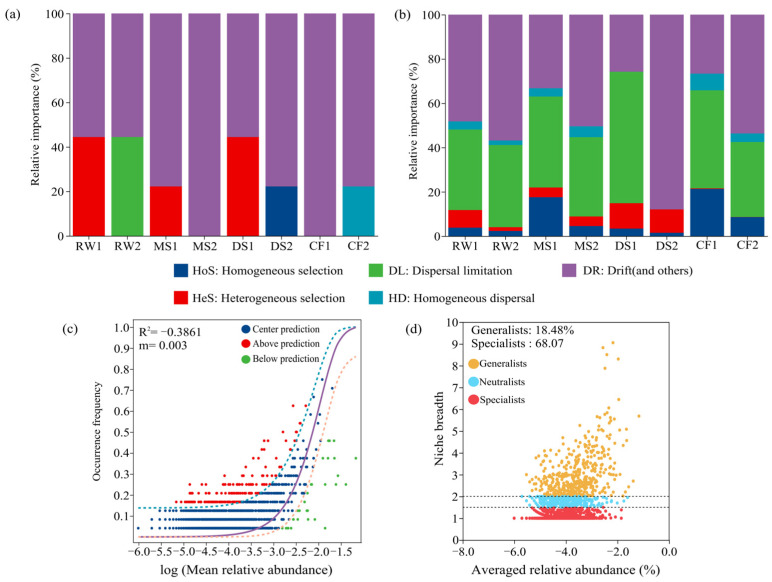
Fungal ecological strategies and community assembly processes across habitat-depth groups. (**a**) Relative importance of fungal community assembly processes based on βNTI. (**b**) Relative contributions of ecological assembly processes estimated by iCAMP. (**c**) Neutral community model (NCM) showing the relationship between fungal taxon occurrence frequency and mean relative abundance. The solid purple curve represents the fitted NCM prediction, and the blue and orange dashed curves indicate the upper and lower 95% confidence limits, respectively. Taxa above, within, and below the prediction range are shown in different colors. (**d**) Niche breadth distribution of fungal taxa classified as generalists, neutralists, and specialists. The two horizontal dashed lines indicate the niche breadth thresholds used to distinguish specialists, neutralists, and generalists. βNTI, beta nearest taxon index; iCAMP, infer community assembly mechanisms by phylogenetic-bin-based null model analysis; NCM, neutral community model; HoS, homogeneous selection; HeS, heterogeneous selection; DL, dispersal limitation; HD, homogeneous dispersal; DR, drift and other undominated processes; RW, reed wetland; MS, meadow steppe; DS, degraded *Suaeda* saline patch; CF, converted farmland; 1, 0–20 cm soil layer; 2, 20–40 cm soil layer.

As shown in Figure 6, fungal communities exhibited distinct ecological strategies and assembly processes across habitat-depth groups. The βNTI-based results showed that drift and other undominated processes contributed the largest proportion in most groups, particularly in MS2 and CF1, where they accounted for nearly all assembly processes. Heterogeneous selection was relatively important in RW1, MS1, and DS1; dispersal limitation was prominent in RW2; homogeneous selection was observed in DS2; and homogeneous dispersal contributed mainly to CF2 (Figure 6a). The iCAMP analysis further indicated that stochastic processes, especially drift and dispersal limitation, dominated fungal community assembly across most habitat-depth groups, whereas homogeneous and heterogeneous selection contributed less and varied among groups (Figure 6b). The neutral community model showed a poor fit to fungal taxon occurrence, with R^2^ = −0.3861 and m = 0.0003, indicating that neutral processes alone could not explain the distribution patterns of fungal taxa (Figure 6c). Niche breadth analysis showed that specialists accounted for 68.07% of fungal taxa, whereas generalists accounted for 18.48%. Specialists were mainly distributed within a narrow niche breadth range, while generalists occupied broader niche ranges and included taxa with higher averaged relative abundance (Figure 6d).

### 3.8. FUNGuild-Based Functional Guilds of Fungal Communities

**Figure 7:**
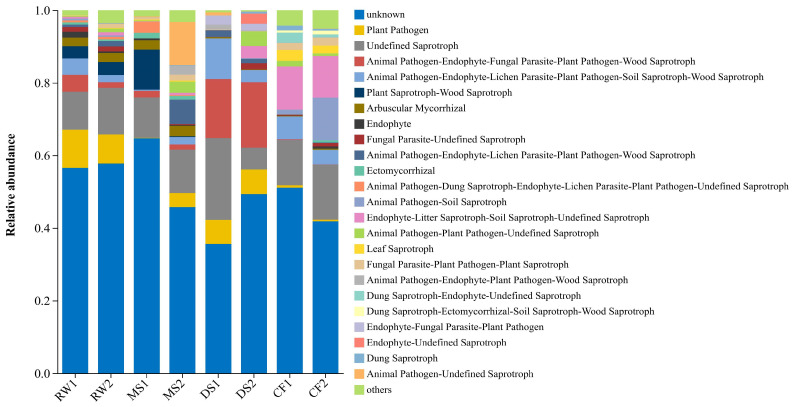
Predicted fungal functional guilds across habitat-depth groups based on FUNGuild annotation. RW, reed wetland; MS, meadow steppe; DS, degraded *Suaeda* saline patch; CF, converted farmland; 1, 0–20 cm soil layer; 2, 20–40 cm soil layer.

As shown in Figure 7, the FUNGuild-based prediction revealed differences in fungal functional guild composition among the habitat-depth groups. The unknown category accounted for the largest proportion across all treatments, with relative abundances of approximately 0.36–0.65; it was relatively higher in MS1, RW1, and RW2, but lower in DS1. In addition to the unknown category, Undefined Saprotroph and Plant Pathogen were the major predicted guilds in most treatments. Undefined Saprotroph occurred at relatively high proportions in DS1, CF2, CF1, RW2, MS1, and MS2, with the highest contribution observed in DS1. Plant Pathogen was mainly detected in the RW, DS, and some MS treatments, whereas its proportion was lower in the CF groups. Differences between soil layers were also observed. MS1 was characterized by a higher proportion of unknown fungi, while MS2 showed an increased contribution of compound guilds such as Animal Pathogen-Undefined Saprotroph. DS1 contained higher proportions of Undefined Saprotroph and Fungal Parasite-Undefined Saprotroph, whereas DS2 showed relatively higher proportions of Plant Pathogen and unknown fungi. In CF1 and CF2, several compound functional guilds were detected in addition to unknown fungi, including Animal Pathogen-Soil Saprotroph and Endophyte-Litter Saprotroph-Soil Saprotroph-Undefined Saprotroph. These results indicate that the predicted fungal functional guilds differed among habitat-depth groups, with saprotrophic, pathogenic, and mixed trophic guilds contributing differently across treatments.

## 4. Discussion

Soil depth and habitat type jointly altered the soil environment in the degraded soda saline–alkali wetland. The degraded *Suaeda* saline patch showed markedly higher pH and EC, whereas the reed wetland maintained higher SWC, SOC, DOC, MBC, and MBN. This pattern indicates that wetland degradation was accompanied by a transition from relatively moist and carbon-rich soils to more alkaline, saline, and nutrient-limited conditions. Similar changes have been reported in degraded wetlands of the Songnen Plain, where wetland degradation altered soil physicochemical properties and fungal community composition [49]. Wetland drainage has also been shown to modify soil pH, EC, moisture, and organic matter, thereby affecting fungal community structure [50]. More broadly, saline soils impose strong environmental constraints on microbial diversity and function through salinity, alkalinity, and nutrient limitation [51]. Fungal richness was highest in CF1 and lowest in DS2, suggesting that surface-converted farmland supported more fungal taxa, while the deeper layer of the degraded saline patch represented a more restrictive habitat. The decrease in richness under severe saline–alkali stress is consistent with evidence that ecosystem degradation can simplify soil fungal communities and reduce their stability and multifunctionality [52]. In this study, Sobs and ACE were more sensitive than Shannon and Simpson indices, indicating that habitat-depth changes primarily affected fungal richness rather than community evenness or dominance. This result suggests that soil depth and degradation influenced the fungal species pool, especially under high salinity, alkalinity, and low nutrient availability.

Ascomycota dominated most habitat-depth groups, especially DS1 and DS2, whereas Basidiomycota and Mortierellomycota were more abundant in converted farmland. This pattern is consistent with studies showing that saline–alkali soil management and environmental gradients can strongly alter fungal community composition, with Ascomycota and Basidiomycota often acting as dominant fungal phyla [53]. In the Hetao Plain, soil factors were also shown to drive microbial community variation across different saline–alkaline soils [54]. The enrichment of Ascomycota in DS soils may reflect the tolerance of some ascomycetous fungi to alkaline, saline, and nutrient-poor environments, whereas the higher proportions of Basidiomycota and Mortierellomycota in CF soils may be related to altered substrate inputs and soil disturbance following agricultural conversion. The LEfSe results further supported the taxonomic differentiation among habitat-depth groups. CF1 was characterized by Mortierellomycota-related taxa, while CF2 was associated with several order-level indicators such as *Thelebolales*, *Tubeufiales*, and *Xylariales*. In contrast, the RW, MS, and DS groups showed different sets of biomarkers from phylum to order levels. Previous work in reed wetlands has shown that soil salinity can shape microbial communities more strongly than plant genotype or geographical distance at a fine spatial scale [55]. Recent coastal wetland studies also suggest that salinity gradients can drive distinct bacterial and fungal community differentiation and assembly patterns [56]. Therefore, the fungal biomarkers observed here indicate that soil depth, salinity–alkalinity, and habitat conversion together generated distinct fungal taxonomic profiles.

The higher relative abundances of Basidiomycota and Mortierellomycota in converted farmland may be related to changes in substrate input, soil disturbance, and nutrient availability after agricultural conversion. Basidiomycota include many decomposer fungi capable of degrading plant residues and complex organic compounds, and their enrichment in CF soils may reflect the influence of crop residues and altered carbon inputs following maize cultivation [57]. Mortierellomycota, particularly members related to *Mortierella*, are commonly associated with nutrient-rich agricultural soils and rhizosphere environments. Some taxa in this group have been reported to participate in phosphorus transformation and organic matter turnover, including the solubilization of poorly available phosphorus, which may partly explain their higher abundance in CF soils with relatively higher AP availability [58]. In contrast, the strong dominance of Ascomycota in DS soils may reflect the ability of many ascomycetous fungi to tolerate saline, alkaline, and nutrient-limited conditions. Their stress tolerance can be associated with resistant cell structures, melanized or thickened cell walls, osmotic adjustment, and the production of stress-resistant spores or hyphae, which may facilitate survival under high pH, high EC, low water content, and limited nutrient availability [59]. Therefore, the contrasting distribution patterns of Ascomycota, Basidiomycota, and Mortierellomycota suggest that fungal taxonomic shifts were closely linked to habitat-specific soil conditions and the ecological functions of different fungal groups.

PCoA, NMDS, and ANOSIM analyses confirmed significant differences in fungal community composition among habitat-depth groups. The clear separation of CF groups from natural wetland and degraded saline patch groups indicates that agricultural conversion produced a distinct fungal community structure. The separation of DS groups also suggests that saline–alkali degradation formed a specific fungal assemblage. Recent research on saline–alkali wetland degradation has shown that bacteria and fungi may display divergent assembly patterns along degradation gradients, highlighting the sensitivity of fungal communities to soil environmental changes [60]. In saline–alkaline soils, vegetation degradation has also been reported to influence fungal β diversity, although the response of fungal communities may differ from that of bacterial communities [61]. CCA and Spearman correlation analyses showed that fungal community composition was associated with multiple soil variables, including pH, EC, SWC, SOC, DOC, MBC, MBN, AN, AP, URE, and ALP. Ascomycota was positively correlated with EC and pH but negatively correlated with most carbon- and nutrient-related variables, suggesting that salinity–alkalinity was closely related to its distribution. In marsh soils, hydrological fluctuation, evaporation-driven salt accumulation, organic matter decomposition, and nutrient transformation are closely linked. The high pH and EC in DS soils, together with low SWC, SOC, DOC, MBC, MBN, AN, and AP, indicate that saline–alkali degradation may constrain microbial biomass, enzyme activity, and the availability of carbon, nitrogen, and phosphorus [62]. Under such conditions, the enrichment of Ascomycota may represent a shift toward stress-tolerant fungal assemblages rather than a direct response to nutrient enrichment. This shift may further affect marsh biogeochemical cycling by changing the fungal groups involved in plant residue decomposition, nutrient mineralization, and plant–soil interactions. Basidiomycota and Mortierellomycota showed different associations with nutrient-related variables, indicating that dominant fungal phyla responded differently to soil environmental gradients. Studies in estuarine wetland soils have also shown that bacterial and fungal community assembly can be regulated by different stochastic processes under heterogeneous environmental conditions [63]. In addition, soil properties rather than plant identity have been identified as important drivers of fungal assembly in dynamic habitats [64]. Taken together, these findings largely support our working hypothesis that soil depth and salinity–alkalinity play important roles in shaping fungal taxonomic structure in degraded soda saline–alkali wetlands. However, the experimental results also indicate that this effect was not solely determined by depth or salinity–alkalinity, but was jointly mediated by nutrient availability, soil moisture, microbial biomass, enzyme activities, and habitat conversion. Thus, the hypothesis was supported in a broader edaphic-gradient context rather than as a single-factor explanation.

The niche breadth analysis showed that specialists accounted for 68.07% of fungal taxa, whereas generalists accounted for 18.48%. This indicates that most fungal taxa had relatively narrow ecological ranges across habitat-depth groups, while a smaller proportion of generalists occupied broader niches. Such a pattern is consistent with strong habitat heterogeneity in saline–alkali wetlands, where differences in water content, salinity, alkalinity, and nutrient availability can restrict the distribution of many fungal taxa. Changes in vegetation and habitat structure have been shown to reconstruct microbial assembly and functionality in coastal salt marshes, suggesting that aboveground habitat transformation and belowground environmental shifts can jointly regulate microbial processes [22]. Soil microbial communities are also closely linked to biogeochemical processes, and changes in microbial assembly may influence carbon and nutrient cycling [65]. The βNTI and iCAMP results indicated that stochastic processes, especially drift and dispersal limitation, contributed substantially to fungal community assembly. However, deterministic selection was also observed in specific groups, such as heterogeneous selection in RW1 and DS1 and homogeneous selection in DS2. This suggests that fungal assembly was not governed by a single ecological process. Instead, stochasticity dominated broadly, while environmental selection became more evident under particular habitat-depth conditions. The poor fit of the neutral community model further indicates that neutral processes alone were insufficient to explain fungal occurrence patterns. Functional differences between major fungal lineages, such as Ascomycota and Basidiomycota, may also contribute to their different responses to soil environments because these groups can differ in enzyme production and decomposition-related functions [65]. Recent wetland studies further emphasize that hydrological regimes and niche partitioning can jointly shape fungal structure and function [66]. Therefore, fungal assembly in this system likely reflected the combined effects of dispersal constraints, ecological drift, habitat filtering, and soil-depth-related environmental heterogeneity.

The FUNGuild results showed that the unknown category occupied a large proportion across all treatments, indicating that many fungal taxa in soda saline–alkali wetlands remain functionally unresolved. This high proportion of unknown taxa may have two possible explanations. First, FUNGuild-based assignments depend on existing taxonomic and ecological information, and many fungal taxa cannot be confidently assigned to functional guilds because of limited database coverage and uneven functional annotation across fungal lineages [67]. Second, the studied soda saline–alkali wetland represents a unique and relatively extreme environment, where high pH, salinity, hydrological fluctuation, and nutrient limitation may support fungal taxa that are poorly described or lack close functional references in current databases [68]. Therefore, the unknown category should not only be regarded as a technical limitation of FUNGuild, but may also reflect the insufficient characterization of fungal diversity in extreme salt-affected wetland ecosystems [69]. Among the annotated guilds, undefined saprotrophs, plant pathogens, and mixed trophic guilds varied among habitat-depth groups, suggesting that shifts in taxonomic composition were accompanied by changes in predicted trophic strategies. Therefore, the functional guild results should be interpreted as predicted ecological tendencies rather than direct functional measurements. Despite this limitation, the variation in predicted guilds provides useful evidence that fungal ecological roles differed among habitats and soil depths. Saprotrophic and mixed trophic guilds may be linked to organic matter decomposition and nutrient cycling, whereas pathogenic guilds may reflect potential plant–soil interactions. Previous work has shown that fungal guilds can act as important integrators linking plant diversity with soil carbon, nitrogen, and phosphorus stocks in dryland ecosystems [70]. In the present study, differences in predicted guilds among RW, MS, DS, and CF groups suggest that wetland degradation and agricultural conversion may alter the potential functional structure of soil fungal communities. Future studies should combine amplicon sequencing with metagenomics, metatranscriptomics, enzyme assays, fungal isolation and cultivation, and seasonal monitoring to verify whether the predicted functional shifts correspond to actual metabolic activity and ecosystem processes in degraded saline–alkali wetlands [71]. In addition, trace metals were not measured in this study, although they may influence soil enzymatic activity and microbial functional status. Elements such as Ni, Cu, Fe, Zn, and Mn can act as enzyme cofactors or affect enzyme activation and inhibition [72]. For example, urease is a Ni-dependent metalloenzyme, and the availability of metal cofactors may influence enzyme activity even when microbial enzyme-producing taxa are present [73]. Therefore, future studies should include trace metal measurements to clarify their potential roles in regulating soil enzyme activity, fungal functional traits, and biogeochemical processes in degraded soda saline–alkali wetlands [74].

## 5. Conclusions

This study showed that soil depth and habitat type jointly shaped fungal communities in a degraded soda saline–alkali wetland. The degraded Suaeda saline patch showed stronger edaphic stress, with higher pH and EC but lower water content, carbon availability, nutrients, and microbial biomass, particularly in the deeper soil layer. Fungal richness was highest in surface-converted farmland and lowest in the deeper degraded saline patch. Fungal community composition differed clearly among habitat-depth groups, with Ascomycota dominating most treatments, especially degraded saline soils, whereas Basidiomycota and Mortierellomycota were more prominent in converted farmland. LEfSe, beta diversity, CCA, and correlation analyses confirmed that fungal community differentiation was closely associated with salinity–alkalinity, soil moisture, nutrient availability, microbial biomass, and enzyme activities. Ecological assembly analyses indicated that stochastic processes, especially drift and dispersal limitation, played major roles in fungal community assembly, while deterministic selection varied among specific habitat-depth groups. Niche breadth analysis suggested a high contribution of specialist taxa, and FUNGuild prediction showed habitat-depth-dependent shifts in saprotrophic, pathogenic, and mixed trophic guilds. Overall, these findings largely support the expectation that soil depth and salinity–alkalinity are key factors structuring fungal communities, although their effects were jointly mediated by nutrient status, water availability, microbial biomass, enzyme activities, and land conversion. This study was based on two soil depths and one sampling period, and functional guilds were inferred from ITS-based taxonomic annotation rather than direct functional measurements. Future studies should combine deeper soil sampling, long-term monitoring, fungal isolation and cultivation, metagenomic, metatranscriptomic, and enzyme-based analyses to verify fungal functional responses. These findings emphasize the importance of considering subsurface soils when assessing belowground biodiversity and provide a basis for the restoration and management of degraded soda saline–alkali wetlands.

## Figures and Tables

**Table 1:** Soil physicochemical properties, microbial biomass, and enzyme activities across habitat-depth groups in a degraded soda saline–alkali wetland.

Soil Variable	RW1	RW2	MS1	MS2	DS1	DS2	CF1	CF2
pH	8.81 ± 0.08 ^b^	8.62 ± 0.27 ^b^	8.71 ± 0.27 ^b^	8.47 ± 0.11 ^b^	10.34 ± 0.30 ^a^	10.30 ± 0.62 ^a^	8.65 ± 0.07 ^b^	8.63 ± 0.50 ^b^
EC (ds·m^−1^)	0.36 ± 0.02 ^c^	0.34 ± 0.02 ^c^	0.36 ± 0.04 ^c^	0.31 ± 0.08 ^c^	1.70 ± 0.07 ^a^	1.75 ± 0.14 ^a^	0.58 ± 0.04 ^b^	0.73 ± 0.05 ^b^
SWC (%)	46.50 ± 1.70 ^a^	40.50 ± 1.60 ^a^	22.17 ± 3.61 ^c^	19.83 ± 2.17 ^cd^	12.83 ± 2.81 ^d^	14.83 ± 1.65 ^d^	26.27 ± 1.96 ^c^	32.17 ± 1.23 ^b^
SOC (g·kg^−1^)	26.17 ± 3.21 ^a^	16.50 ± 1.87 ^b^	17.52 ± 2.93 ^b^	11.17 ± 1.23 ^c^	6.51 ± 1.30 ^a^	4.27 ± 0.50 ^d^	16.50 ± 0.70 ^b^	11.47 ± 0.65 ^c^
SBD (g·m^−3^)	0.96 ± 0.04 ^d^	0.85 ± 0.03 ^d^	1.01 ± 0.05 ^d^	1.14 ± 0.05 c	1.25 ± 0.11 ^b^	1.25 ± 0.05 ^b^	1.34 ± 0.04 ^b^	1.57 ± 0.06 ^a^
AN (mg·kg^−1^)	192.73 ± 18.94 ^a^	115.50 ± 13.1 ^b^	129.70 ± 22.21 ^b^	83.50 ± 10.18 ^c^	46.43 ± 11.52 ^d^	27.83 ± 5.13 ^d^	225.51 ± 10.30 ^a^	135.52 ± 7.31 ^b^
AP (mg·kg^−1^)	13.27 ± 1.96 ^b^	8.50 ± 0.89 ^c^	9.07 ± 1.69 ^c^	6.03 ± 0.86 ^d^	4.27 ± 1.20 ^d^	2.60 ± 0.56 ^d^	28.40 ± 2.15 ^a^	12.50 ± 1.30 ^b^
DOC (mg·kg^−1^)	388.68 ± 16.38 ^a^	345.92 ± 17.26 ^b^	370.31 ± 9.04 ^a^	337.11 ± 2.73 ^b^	211.80 ± 3.70 ^d^	198.16 ± 7.07 ^d^	256.99 ± 4.2 ^c^	238.29 ± 12.02 ^c^
MBC (mg·kg^−1^)	1865.06 ± 51.86 ^a^	1537.30 ± 36.54 ^b^	1402.30 ± 13.97 ^b^	1297.16 ± 38.21 ^c^	481.49 ± 5.66 ^d^	245.66 ± 38.69 ^d^	1241.80 ± 24.36 ^c^	770.18 ± 97.22 ^d^
MBN (mg·kg^−1^)	256.11 ± 6.52 ^a^	256.11 ± 6.52 ^a^	156.56 ± 6.76 ^b^	139.77 ± 3.80 ^b^	68.24 ± 5.18 ^d^	46.13 ± 2.71 ^d^	118.42 ± 1.37 ^c^	100.64 ± 1.92 ^c^
URE (mg·g^−1^)	102.08 ± 53.28 ^a^	71.93 ± 15.68 ^ab^	70.27 ± 5.55 ^ab^	70.21 ± 2.74 ^ab^	34.74 ± 16.70 ^b^	22.51 ± 6.07 ^b^	63.22 ± 9.08 ^ab^	74.46 ± 11.00 ^ab^
ALP (mg·g^−1^)	158.61 ± 62.57 ^ab^	116.20 ± 3.33 ^b^	125.57 ± 14.06 ^b^	128.46 ± 36.22 ^ab^	157.93 ± 31.13 ^ab^	217.90 ± 32.53 ^a^	172.76 ± 9.01 ^ab^	124.88 ± 26.48 ^b^

Note: Data are presented as the mean ± standard deviation (SD) (*n* = 3). Different lowercase letters indicate significant differences among habitat-depth groups according to Tukey’s HSD test at *p* < 0.05. Abbreviations: RW, reed wetland; MS, meadow steppe; DS, degraded *Suaeda* saline patch; CF, converted farmland; 1, 0–20 cm soil layer; 2, 20–40 cm soil layer; EC, electrical conductivity; SWC, soil water content; SOC, soil organic carbon; SBD, soil bulk density; AN, available nitrogen; AP, available phosphorus; DOC, dissolved organic carbon; MBC, microbial biomass carbon; MBN, microbial biomass nitrogen; URE, urease activity; ALP, alkaline phosphatase activity.

**Table 2:** Fungal alpha diversity indices across different habitat-depth groups.

Treatments	Sobs	Shannon	Ace	Simpson
RW1	186.67 ± 45.34 ^bc^	3.79 ± 0.72 ^a^	190.37 ± 84.80 ^bc^	0.056 ± 0.033 ^a^
RW2	204.33 ± 24.99 ^bc^	4.03 ± 0.32 ^a^	209.12 ± 28.91 ^bc^	0.063 ± 0.050 ^a^
MS1	155 ± 32.08 ^bc^	4.03 ± 0.17 ^a^	156.96 ± 33.44 ^bc^	0.037 ± 0.004 ^a^
MS2	208.67 ± 76.54 ^bc^	4.16 ± 0.54 ^a^	212.37 ± 78.55 ^bc^	0.047 ± 0.009 ^a^
DS1	110.33 ± 47.18 ^c^	2.75 ± 0.42 ^a^	115.35 ± 51 ^c^	0.136 ± 0.075 ^a^
DS2	86 ± 17.29 ^c^	2.57 ± 0.48 ^a^	87.24 ± 48.59 ^c^	0.083 ± 0.144 ^a^
CF1	423.33 ± 28.36 ^a^	5.55 ± 0.36 ^a^	622.76 ± 132.16 ^a^	0.144 ± 0.055 ^a^
CF2	267 ± 42.61 ^b^	3.44 ± 0.32 ^a^	274.57 ± 172.38 ^b^	0.115 ± 0.029 ^a^

Note: Data are presented as the mean ± standard deviation (SD) (*n* = 3). Different lowercase letters within the same column indicate significant differences among habitat-depth groups according to Tukey’s HSD test at *p* < 0.05. Abbreviations: RW, reed wetland; MS, meadow steppe; DS, degraded Suaeda saline patch; CF, converted farmland; 1, 0–20 cm soil layer; 2, 20–40 cm soil layer.

## Data Availability

The original contributions presented in this study are included in the article. The raw sequencing data have been deposited in the NCBI Sequence Read Archive under BioProject accession number PRJNA1472827.

## Abbreviations

The following abbreviations are used in this manuscript:

EC	Electrical conductivity
SWC	Soil water content
SOC	Soil organic carbon
SBD	Soil bulk density
AN	Available nitrogen
AP	Available phosphorus
DOC	Dissolved organic carbon
MBC	Microbial biomass carbon
MBN	Microbial biomass nitrogen
URE	Urease activity
ALP	Alkaline phosphatase activity
OTU	Operational taxonomic unit
ITS	Internal transcribed spacer
PCoA	Principal coordinate analysis
NMDS	Non-metric multidimensional scaling
CCA	Canonical correspondence analysis
LEfSe	Linear discriminant analysis effect size
LDA	Linear discriminant analysis
βNTI	Beta nearest taxon index
iCAMP	Infer community assembly mechanisms by phylogenetic-bin-based null model analysis
NCM	Neutral community model
